# Impact of a resilience-building energy management intervention for people with systemic sclerosis: a mixed methods study

**DOI:** 10.1093/rap/rkae040

**Published:** 2024-03-08

**Authors:** Yen T Chen, Alexandra E Harper, Tiffany Phanhdone, Mary Alore, Sheri Hicks, Adam Pape, Gina M Jay, Shannen Bolde, Jennie Feldpausch, Timothy C Guetterman, Dinesh Khanna, Susan L Murphy

**Affiliations:** Division of Rheumatology, Department of Internal Medicine, University of Michigan, Ann Arbor, MI, USA; Department of Physical Medicine and Rehabilitation, University of Michigan, Ann Arbor, MI, USA; Department of Physical Medicine and Rehabilitation, University of Michigan, Ann Arbor, MI, USA; Rush University Medical Center, Chicago, IL, USA; Division of Rheumatology, Department of Internal Medicine, University of Michigan, Ann Arbor, MI, USA; Division of Rheumatology, Department of Internal Medicine, University of Michigan, Ann Arbor, MI, USA; Division of Rheumatology, Department of Internal Medicine, University of Michigan, Ann Arbor, MI, USA; Department of Physical Medicine and Rehabilitation, University of Michigan, Ann Arbor, MI, USA; Department of Physical Medicine and Rehabilitation, University of Michigan, Ann Arbor, MI, USA; Division of Rheumatology, Department of Internal Medicine, University of Michigan, Ann Arbor, MI, USA; Department of Family Medicine, University of Michigan, Ann Arbor, MI, USA; Division of Rheumatology, Department of Internal Medicine, University of Michigan, Ann Arbor, MI, USA; Division of Rheumatology, Department of Internal Medicine, University of Michigan, Ann Arbor, MI, USA; Department of Physical Medicine and Rehabilitation, University of Michigan, Ann Arbor, MI, USA

**Keywords:** systemic sclerosis, fatigue, symptom management, peer health coach, resilience, well-being, mixed methods study

## Abstract

**Objectives:**

People with SSc often experience fatigue, which significantly affects daily life functioning and quality of life. We aimed to explore participants’ experiences of a peer health–coached resilience-building energy management to enhance well-being (RENEW) intervention on symptoms and well-being and to use mixed methods to compare how SSc duration influenced the experiences of participants who had clinically significant fatigue improvement *vs* those who did not.

**Methods:**

Semi-structured interviews were conducted with 21 participants from the parent clinical trial randomized to the RENEW intervention. Data were analysed using the rigorous and accelerated data reduction technique combined with thematic content analysis. A mixed methods approach used a joint display to identify themes related to the impact of SSc duration on fatigue improvement status. Participants were categorized into short/improvement, short/limited improvement, long/improvement, and long/limited improvement.

**Results:**

Our team generated four themes: participant and peer health–coach relationship, physical and psychological well-being improvement, need for a tailored approach and easy program access through technology. Mixed methods analysis revealed that, regardless of SSc duration, participants with improved fatigue reported increasing self-awareness of SSc-related symptoms and learning coping strategies to manage fatigue. Participants in the short/improvement group reported preferences for slower pacing of the program and pairing with a coach with similar symptom severity. Participants in the long/limited improvement group sought SSc-specific symptom management information.

**Conclusion:**

Incorporating peer health coaches and technology is beneficial for self-management interventions for people with SSc. Future tailoring of RENEW based on SSc duration and symptom severity is needed.

**Clinical trial registration number:**

clinicatrials.gov, NCT04908943.

## Introduction

Key messages

- Peer health coaching and web-based/mobile technology are beneficial intervention components for people with SSc.
- Participants with short SSc duration and improved fatigue post-intervention had specific preferences for health coaching.
- Participants with long SSc duration and limited fatigue improvement wanted more SSc-specific symptom management information.

SSc, a rare and chronic autoimmune disease characterized by fibrosis and vascular abnormalities, can affect multiple organ systems [1] and result in significant symptom burden that diminishes quality of life [2–6]. Fatigue is among the most prevalent and debilitating symptoms, affecting >70% of people with SSc [7], significantly hindering their ability to perform daily tasks [8–10], maintain employment [9, 11, 12] and fulfil life roles [8, 9, 13]. Unfortunately, addressing fatigue in SSc remains an unmet clinical need [14]. A recent study examined the effects of a self-management program, including fatigue management for people with SSc, but failed to show significant improvements in fatigue levels [15].

Emerging evidence suggests that a structured program led by a peer health coach might be important for improving the management of chronic diseases [16]. Self-management programs incorporating peer coaching demonstrated promise in improving physical and psychological symptoms in people with chronic conditions [17–19]. Non-pharmacological interventions like physical activity [20–22] and activity pacing [23–25], which have shown benefits for people with SSc and other rheumatic diseases, warrant consideration. Additionally, resilience, referring to an individual’s ability to ‘bounce back’ from adversity and overcome challenges [26], has been recognized as a protective factor in interventions by rheumatology researchers, enabling individuals to navigate and respond to new challenges that may emerge during the course of their diseases [27, 28]. Thus the development of a peer-coached fatigue intervention program that promotes health behaviours and resilience for individuals with SSc may fulfil this unmet clinical need.

Our research team, in collaboration with SSc patient partners, developed a 12-week resilience-building energy management to enhance well-being (RENEW) program. This intervention draws upon insights from previous literature [17–25], theories of self-efficacy [29] and positive psychology [30, 31]. In the RENEW program, participants worked with a trained peer health coach with SSc, integrating healthy behaviours into their daily routine. Together, they set and monitored health goals, with the peer health coach providing feedback as needed. In the quantitative phase, we conducted a randomized controlled trial (RCT) to assess the effectiveness of the RENEW program on symptoms and resilience in participants with SSc [32].

The quantitative findings indicated that the intervention program was effective for reducing fatigue, particularly in those with short SSc duration [32]. In the early disease, SSc often presents with oedematous skin involvement, RP and joint involvement, which contribute to a high disease burden and increased fatigue [2], particularly with diffuse cutaneous SSc, which may respond favourably to interventions [33]. In contrast, as the disease progresses, manifestations such as telangiectasia, calcinosis, lower gastrointestinal involvement and sicca symptoms become more prominent [34]. While these later-stage manifestations remain significantly burdensome for patients, they pose challenges in terms of improvement with treatment and may have fewer available therapeutic options. As such, the role of SSc duration warranted further exploration. Expanding on the quantitative results, this study conducted semi-structured interviews with participants in the RENEW program. This study had two objectives: to explore participants’ experiences regarding the effects of RENEW on their symptoms and well-being using qualitative data and to use mixed methods to compare how SSc duration [short (diagnosed with SSc for ≤5 years) or long (diagnosed with SSc for >5 years)] influenced the experiences of participants who had clinically meaningful improved fatigue [35] (i.e. ≥3 point change from baseline to 12 weeks) *vs* those who did not.

## Methods

### Study design

This study used an explanatory sequential mixed methods design [36, 37]. Findings from the quantitative analysis have been published [32]. Qualitative data were collected after participants completed the intervention to elucidate experiences and examine how participation in RENEW influenced symptoms and well-being. A mixed methods approach, which integrates the quantitative and qualitative results using a joint display [38], was used to examine themes related to the impact of short or long SSc duration on fatigue. This strategy enhances the strengths of both methods and reduces weaknesses that can arise from collecting and analysing either quantitative or qualitative data separately [36]. This study was approved by the University of Michigan Institutional Review Board (HUM00195121).

### Participants

**Figure 1.**
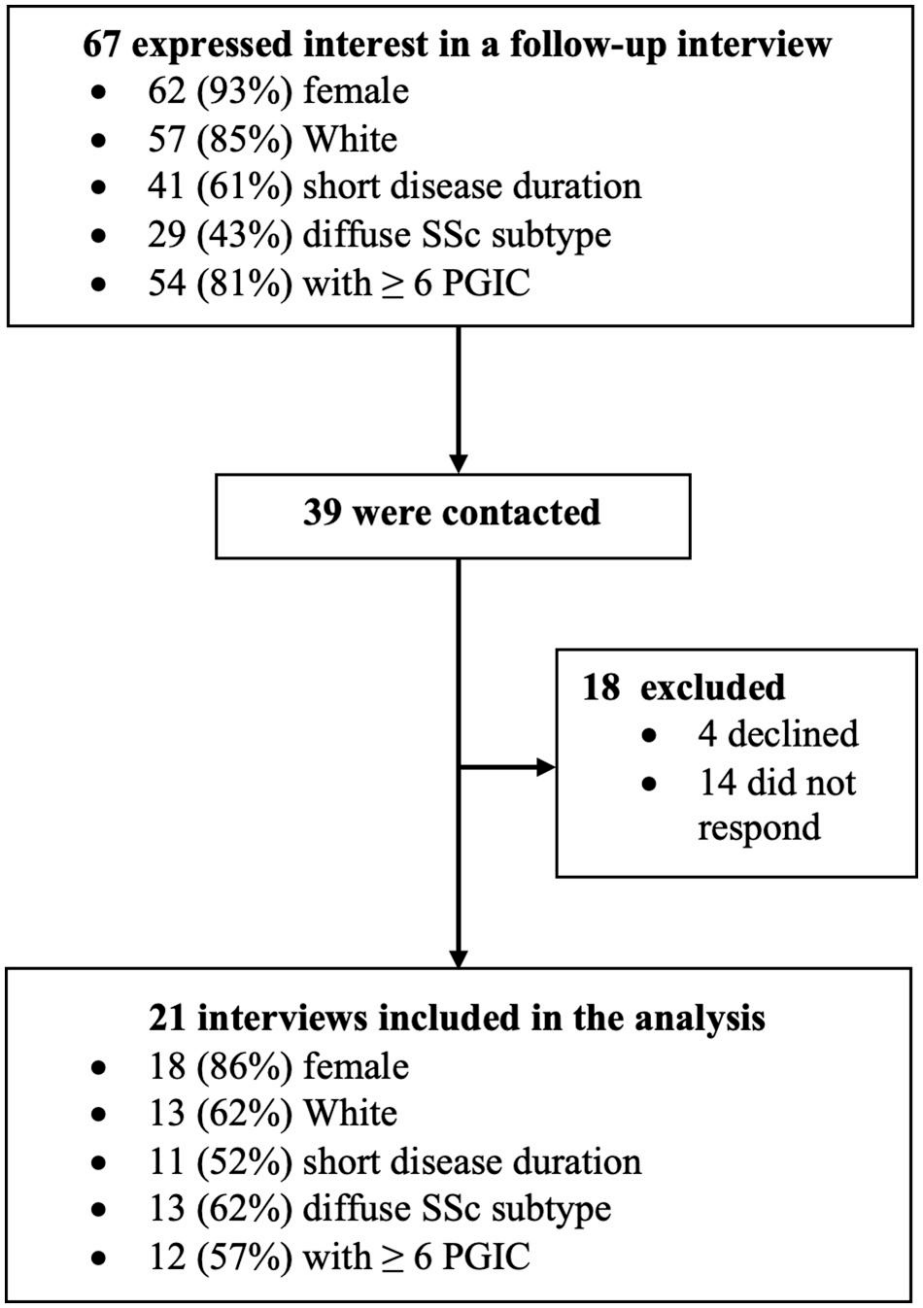
Participant flowchart

Recruitment and participants’ inclusion and exclusion criteria are reported elsewhere [32]. All participants provided informed consent electronically. At the post-intervention assessment, respondents from the RENEW group (*n* = 115) indicated their willingness to participate in a brief interview, and a subsample of these participants was contacted and invited to participate (Fig. 1). Twenty-one participants were purposefully invited to participate based on sex, race/ethnicity, SSc duration (i.e. ≤5 years or >5 years) and Patient Global Impression of Change (PGIC) [39] score (i.e. ≥6 or ≤3) to capture a variety of experiences. We continued interviewing participants until we had representation from individuals historically underrepresented in SSc research (i.e. male, non-White) and data saturation was reached (*n* = 21).

### Intervention

The RENEW intervention is an online 12-week resilience-building program facilitated by peer health coaches who also have SSc. It comprises seven modules that address a range of health topics aimed at alleviating symptoms and enhancing well-being. Over the 12 weeks, peer health coaches conducted nine 15–30 min virtual sessions with participants to assist with goal setting and modification and to provide feedback and support [32].

### Data collection

Semi-structured interviews took place from August 2021 to June 2023. These interviews (≈30 min) included six open-ended questions (Table 1) developed by the research team, including SSc patient partners. Interviews were conducted and audio recorded by either Y.C. or G.J. on Zoom then transcribed verbatim.

**Table 1.** Interview questions.

1. Overall, do you think participating in the program helped you manage your scleroderma? Probing questions In what ways did the program help you: With symptoms (like fatigue, pain, sleep disturbance) With your physical function or abilities (like mobility of joints, walking, holding objects) With negative mood (depressed feelings)

2. Is there any way that the program could be changed to better suit your needs? Probing questions What about: More or less interaction with health coach More guidance in goal setting Content that was missing Offered in a different way

3. What was most challenging about participating in the program? Probing questions What about: Technology issues (e.g. calls with health coach) Tracking not easy for my set goal Ability to make progress Health issues

4. What was the best aspect of this program for you? Probing questions What about: Content Program designed specifically for people with scleroderma Health coach interaction

5. Can you provide an example from your experience of how you found this program helpful?
6. If you could tell the developers of this program anything you wanted, what would you want to let them know? This could be something that would be useful to improve the program or something that was very good about your experience with the program.

### Data analyses

#### Qualitative data analysis

Qualitative data were analysed using a codebook approach that combined the Rapid and Rigorous Qualitative Data Analysis (RADaR) technique [40] with reflexive thematic analysis. This approach sought to establish accuracy among multiple coders, as with traditional coding reliability approaches, and to iteratively identify themes from codes generated from interview data, as with reflexive thematic analysis [41–44]. Our goal was to ensure a rigorous, systematic, efficient and iterative process for a team of analysts with mixed research experience.

First, Y.C., A.H., T.P. (a patient partner) and S.M. (a senior researcher) familiarized themselves with the data by reading the transcripts multiple times. Second, three coders (Y.C., A.H. and T.P.) conducted data reduction to eliminate content unrelated to the research question. Third, these coders independently conducted open coding to identify relevant codes representing important concepts and ideas related to participant experiences in RENEW and to create a preliminary codebook. Codes were not established a priori. Once initial codes were generated from the reduced data, Y.C. used the preliminary codebook to code the first 25% of the data and A.H. and T.P. independently coded the second 25%. Results were compared and discussed as a team with S.M. to reach a consensus on the refined codebook before progressing. Y.C. then coded the remaining transcripts, which were reviewed for agreement by A.H. and T.P. Trends in the data were discussed and summarized into categories during team meetings. Throughout this iterative process we critically examined the identified categories, ultimately leading to the generation, refinement and naming of key themes. We gathered and processed feedback at each analysis step and documented all qualitative analysis procedures to ensure transparency. The codebook and qualitative findings were reviewed and approved by community insiders—M.A., S.H., A.P. (RENEW health coaches) and D.K. (a world-renowned SSc rheumatologist)—during a member-checking session.

#### Mixed methods analysis

To integrate the qualitative and quantitative findings, we created a joint display, a visual strategy that can be used to bring together different types of results for analysis and interpretation [36, 38]. We sought to compare the experiences of participants who had clinically significant improvement in fatigue [35] and those who did not by SSc duration groups. Accordingly, participants were categorized into four groups: short/improvement, short/limited improvement, long/improvement and long/limited improvement. Individual cases were presented as exemplars to illustrate these findings.

## Results

A total of 21 participants (18% of all participants who did/finished the RENEW intervention) completed interviews. The sample’s mean age was 57 years (s.d. 14). There were 18 females, 13 with the diffuse SSc subtype and 11 with short SSc duration (≤5 years) [33]. Twelve participants had a PGIC score ≥6 (i.e. better and a definite improvement) and 11 participants showed a 3-point improvement in fatigue from baseline to 12 weeks (considered clinically significant) [35]. Table 2 presents participant characteristics.

**Table 2.** Sample characteristics by disease duration (*n* = 21).

SSc duration	I.D.	Fatigue change^a^	PGIC score	Age (years)	Sex	Race	SSc subtype
Short SSc duration (≤5 years)	27	−14.4	7	40	F	White	Limited
	26	−12.5	6	65	F	Asian & other	Limited
	96	−12.2	7	58	F	African American	Diffuse
	108	−12.0	6	63	M	White	Diffuse
	4	−10.3	6	31	F	Asian and other	Diffuse
	135	−9.8	6	59	F	African American	Diffuse
	93	−8.9	6	51	M	White	Diffuse
	73	−8.2	7	61	F	White	Diffuse
	131	−3.6	3	87	F	White	Diffuse
	107	−1.8	3	44	F	White	Limited
	83	−1.6	6	54	F	African American	Overlap
Long SSc duration (>5 years)	34	−16.1	7	47	F	White	Limited
	142	−11.0	6	35	F	African American	Diffuse
	63	−2.8	3	56	F	White	Diffuse
	47	−2.6	3	78	F	White	Overlap
	65	−2.2	3	63	F	White	Limited
	115	0	3	58	F	White	Limited
	144	1.7	3	72	M	White	Diffuse
	1	1.8	2	69	F	White	Diffuse
	24	5.3	6	40	F	African American	Diffuse
	29	17.4	1	59	F	African American	Diffuse

a To assess the change in fatigue, we calculated the difference between the fatigue score at baseline and the score at 12 weeks. Negative values indicate a favourable change, suggesting an improvement or decrease in fatigue. PGIC: 1 = no change (or condition has gotten worse); 2 = almost the same, hardly any change at all; 3 = a little better, but no noticeable change; 4 = somewhat better, but the change has not made any real difference; 5 = moderately better, and a slight but noticeable change; 6 = better and a definite improvement that has made a real and worthwhile difference; 7 = a great deal better and a considerable improvement that has made all the difference.

### Results of qualitative analysis

**Figure 2.**
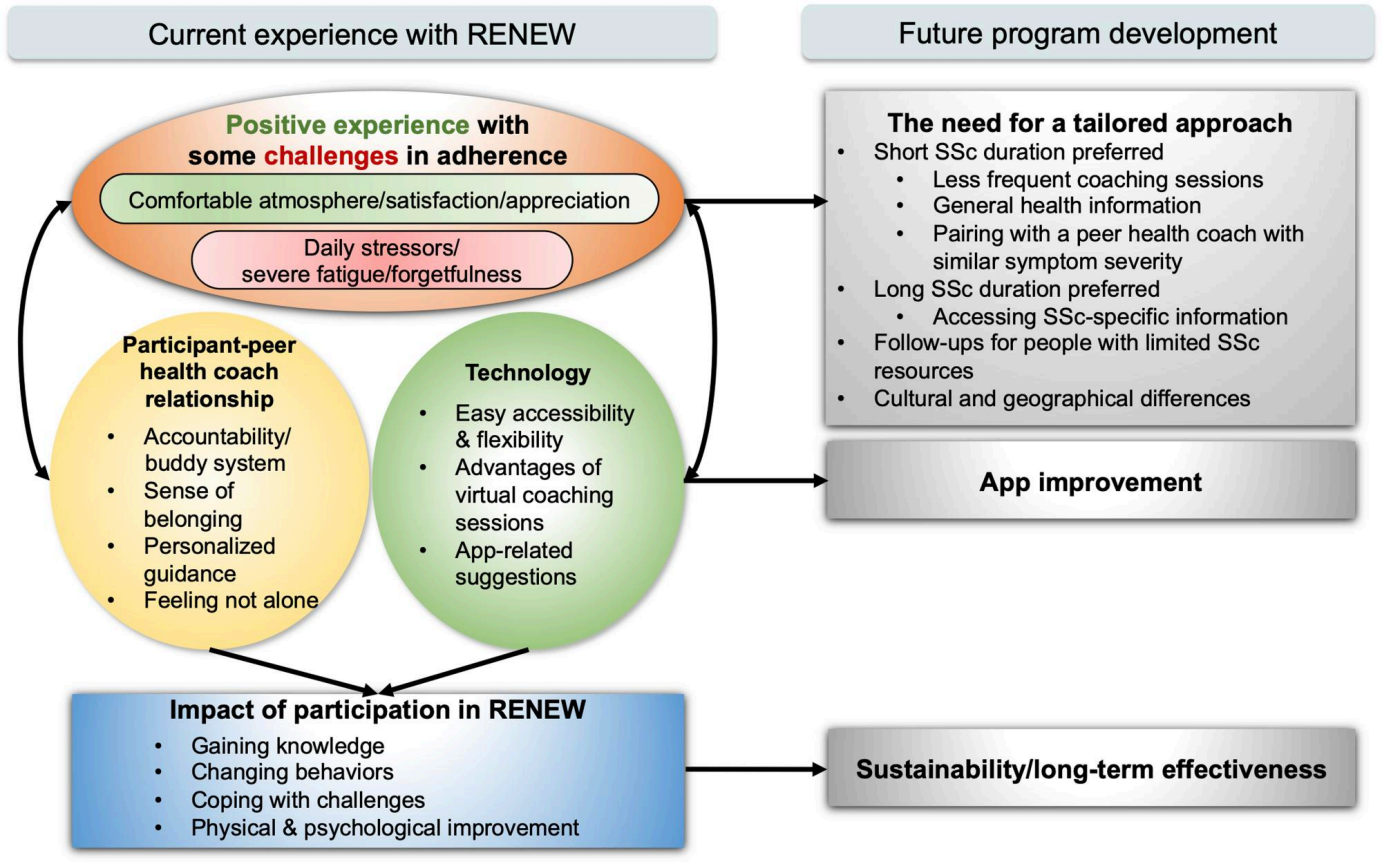
A conceptual model representing the participants’ experiences in RENEW

Our team generated four themes from the qualitative data analysis: participant–peer health coach relationship, physical and psychological well-being improvement, need for a tailored approach and easy program access through technology. A conceptual model visualizing these themes is depicted in Fig. 2. Themes are presented below with exemplar quotes in Table 3.

**Table 3.** Representative quotes for each theme.

Category	Example quote
Theme: Participant–peer health coach relationship	
Accountability/‘buddy’ system	The buddy aspect was very helpful. It’s nice to have someone there who understands…who has hints and things that they’ve tried and that worked for them. (ID #29)
	When my coach said, ‘Congratulations you’re really hit that one out of the park this week, you’re doing great, keep it up’. Just the motivation behind it…It was wonderful. (ID #34)
Sense of belonging	Somebody who’s been there and can understand the challenges that you’re going through. (ID #34)
	You have someone, I mean, who is kind of in the ‘same boat’ you are…They could offer suggestions, so that was really valuable to me, that experience. (ID #29)
Personalized guidance/collaborative problem-solving	My coach helped me be realistic with it. She helped me be able to come up with goals that I can maintain. (ID #83)
	I really enjoyed being able to connect with somebody…Be able to throw ideas at one another of these things that were helpful for me. (ID #34)
Feeling not alone	Having the support and encouragement from my health coach are very important. It showed me that it’s okay to face challenges, and I’m not alone. (ID #63)
Theme: Physical and psychological well-being improvement	
Physical improvement	It helped with the fatigue definitely because some of the tools that I use kind of forced me to be more present, more aware, intentional. (ID #135)
	The modules they had in place helped me to manage primarily my energy, how I was functioning on the days that I felt well. (ID #96)
	It helped me especially with sleep. (ID #107)
	I have increased my endurance with exercising. I think that’s tremendous. (ID #1)
Psychological improvement	I felt emotionally better, I felt a little bit more connected with someone. (ID #73)
	I was starting to feel good and having the modules and having someone to talk to every week helped me to motivate and get myself better and doing all of the right things. (ID #108)
	It helped a lot with anxiety. Like to bring anxiety down. (ID #26)
	It helped me with my stress management. That was a good thing. It helped having a partner to walk me through it. (ID #29)
	I’m a very goal-oriented person. I love to set a goal and be able to check it off or scratch something off my list, so for me it was great. (ID #34)
	I keep working on it, and I think that’s because of the RENEW program. I really want to have things set up to make the things I learned in the program a part of my life, so I’m still plugging away, and not giving up. (ID #29)
Theme: Need for a tailored approach	
Frequency of coaching session	The 6 initial every week, and then every other week, I think that’s sufficient. (ID #144)
	I did a little better when we were meeting weekly. (ID #65)
	When it went every two weeks, I felt a real lack. (ID #47)
	I would have preferred having a little more time because, for example, when you’re listening to a certain program to kind of learn whether that’s going to help you go to sleep better it takes a few evenings to kind of practice it to see if it is going to be effective. (ID #73)
Follow-ups for people with limited SSc resources	I live in a small town, and there aren’t resources around me. So the ability to connect to someone who would understand what I was saying was critical for me because I was feeling so isolated. (ID #26)
	It would be great if we could have some follow-up afterwards…Just to have a list of people that maybe might be closer in proximity to us or something too here in the Midwest. (ID #73)
	I guess one of the drawbacks being somebody that’s not in and around the Michigan scene. Obviously, the amount of additional supports or specialists, and that type of stuff. (ID #93)
Seek SSc-specific information	Giving you suggestions on how you could improve your breathing. (ID #115)
	How to even take care of those things like with the ulcers? (ID #24)
	Things that could help with the hands, so that you can have more strength and ability to pick up things. (ID #115)
Cultural and geographical differences in healthcare	It’s like the disconnect between what’s available here in New Zealand or even in Australia. I guess some of the terminology around some of that as well. So like for here, obviously, we use the mycophenolate as the main understanding what it is. But most other people, they tend to use it as their brand name, CellCept. So it’s like just getting my head around that initially was a little bit of a ‘so what are you talking about?’ (ID #93)
Theme: Easy program access through technology	
Easy accessibility and flexibility	I really enjoyed the ability to go online and look at information for myself and take my time to digest some of that and follow some of the links and kind of go down the rabbit holes with some of it. (ID #34)
Advantages of virtual coaching sessions	Zoom is nice because then it makes it a little bit more real. It’s more as a phone call…just to be able to see that person it’s nice. (ID #63)
	Zoom I think worked out well, and it worked into our schedule. (ID #27)
App-related suggestions	Just little things that they could tweak about the app. It was really the user friendliness of it. (ID #96)
	The app itself was very clunky…Like when there was a place to track things. I found that I wasn’t using that app because it just wasn’t…like sometimes the goal, my goals didn’t fit with the way that I could input them, and there wasn’t much flow. (ID #107)
	I’m just not real computer savvy and like when I talked to my coach about it, I was so frustrated with that app…maybe a little more training on the app, for older people? I have to have a little training on anything that’s got to do with the smartphone. (ID #65)

#### Participant–peer health coach relationship

Peer health coaches were frequently mentioned when discussing what participants thought was the best part of the program. Participants perceived the peer health coaches to be a ‘buddy’ who could provide helpful tips from their own lived experiences with SSc and accountability for achieving goals and help maintain motivation throughout the program. Connecting and identifying with someone who also had SSc and understood the challenges of the disease fostered a sense of belonging where they previously felt isolated. Participants felt like they were in the ‘same boat’ and found this support to be irreplaceable. They expressed gratitude for the emotional and practical support provided by their peer health coaches. Additionally, personalized guidance and collaborative problem-solving were key aspects of the positive participant–coach relationship.

#### Physical and psychological well-being improvement

Many participants reported adopting health behaviours and learning coping strategies during RENEW, ultimately resulting in improved physical and psychological well-being. Physically, participants noted improvements that included reduced fatigue, increased energy levels, enhanced sleep quality and improved exercise endurance. In terms of psychological improvements, participants noted that RENEW positively impacted their mood and motivation to engage in healthy behaviours as well as reduced anxiety and stress levels. Importantly, achieving their health goals in RENEW boosted their confidence, which in turn enhanced their resilience in dealing with everyday life challenges.

#### Need for a tailored approach

Participants expressed diverse preferences regarding the frequency of peer health coaching sessions. While some were content with the current frequency, others favoured more frequent sessions to enhance their engagement and support. Nonetheless, others preferred less frequent sessions due to individual reasons, such as a desire for extra time to work on their goals due to conflicts or time constraints. Many participants desired ongoing support post-program, particularly for those with limited access to SSc resources. A few participants reported either minimal or no improvement in their symptoms despite diligently following the program recommendations. These participants sought more detailed information on managing specific aspects of their SSc symptoms, such as breathing difficulties, digital ulcer care and hand exercises to improve strength. Notably, international participants emphasized the importance of information that extended beyond an American perspective. They cited a disconnect between available resources and the terminology used in their countries compared with the USA.

#### Easy program access through technology

Participants found it convenient to access the RENEW information in one place through the website or the app on their mobile device. The flexibility to learn at their own pace was appreciated. Moreover, virtual coaching sessions offered advantages of remote participation and flexible scheduling. However, participants identified areas for improvement, particularly in terms of the app’s user-friendliness and functionality, as it was challenging to set goals and track behaviours effectively. A few participants, particularly those less familiar with technology or older in age, struggled to navigate the app and suggested some guidance and training in using the app.

### Results of mixed methods analysis

Table 4 presents a comparison of quantitative and qualitative results on fatigue improvement by SSc duration using a joint display to illustrate the findings of the mixed methods analysis. Our analysis revealed a common thread across participants in all groups (short/improved, short/limited improvement, long/improved, long/limited improvement): they found great value in having a peer health coach to guide them through RENEW. Regarding the impact of participation, participants in the short/improved and long/improved groups described an increase in self-awareness of SSc-related symptoms. They learned to manage their fatigue better by activity pacing, avoiding overexertion and incorporating relaxation and mindfulness into their routines. Even those in the short/limited improvement group noted feeling less frustrated and were more able to complete their daily activities. On the other hand, participants in the long/limited improvement group still struggled with severe SSc symptoms despite their participation in RENEW.

**Table 4.** Joint display comparing quantitative and qualitative findings on fatigue improvement by SSc duration (*N* = 21)

SSc duration Groups	Fatigue groups	Qualitative findings
Theme: Participant and peer health coach relationship		
Short SSc duration (*n* = 11)	Improved	**Accountability** You had the accountability which I loved. Because I was like, oh man I have to talk to my coach. I got to make sure I get my stuff done, this is good for me, and it was just an extra push which I think people need. (ID #27)
	Limited improvement	**Social interaction** The coach interaction is the best part of the program. (ID #107)
Long SSc duration (*n* = 10)	Improved	**Support from someone with SSc is helpful** Because it was somebody with scleroderma. So, I found it to be more helpful in a way because that person she understood more what I was going through. So she could relate to me. (ID #142)
	Limited improvement	**Importance of connecting with someone who also has SSc** I love my coach…because she actually has the disease…that was really important because then we feel like we’re comfortable with those people. (ID #24)
Theme: Physical and psychological well-being improvement		
Short SSc duration (*n* = 11)	Improved	**Increased self-awareness/skills learned** I was doing things that I consciously realized I have to have a new pace that I’m going at now and not trying to still be the old person that I used to be and learning to relax more. (ID# 73)
	Limited improvement	**No physical change but reduced frustration and improved coping** I don’t know that anything was reduced, but it helped me feel less frustrated…just sort of that help me cope with it in a way that I felt like I was more functional, even if it wasn’t like I actually was physically feeling better. (ID #107)
Long SSc duration (*n* = 0)	Improved	**Increased self-awareness in activity pacing** Just be mindful of not overdoing it in a day; if you’re going to do something for a couple of hours, you may need to sit and rest for 10 or 15 minutes, or even half an hour. (ID #34)
	Limited improvement	**Severe symptoms did not improve or change** My symptoms are very strong and if you can see my hands—the pain is still there, the lack of sleep is still a problem. You know, so those things have not gotten better. (ID #1)
Theme: Need a tailored approach		
Short SSc duration (*n* = 11)	Improved	**Satisfied with the program** For the content, I mean they really covered all bases. I’ve really enjoyed myself in this program…I mean, there’s really nothing I could see that would need improvement. (ID #27) **Slower pacing of the program** When you’re newly diagnosed with scleroderma and you’re getting to all these doctor appointments…it’s just kind of a lot to have on your schedule and in front of you. So, it might even be that for me personally, a little bit slower pace, maybe. I don't know that once a month would have been quite enough but maybe biweekly. (ID #73) **Pairing with similar symptom severity coach** Like my health coach, I feel like her symptoms are worse than mine. And she’s not doing as much physical exercise as me, so like when we were setting the goals and going through what I can do every week to help my symptoms, she couldn’t help a lot…Maybe for the health coach, if we could like, divide people into different degrees of the severeness of their disease. So, I could pair up with somebody who has a milder symptom and hear how that person’s helping themselves and other patients or participants. (ID #4)
	Limited improvement	**Challenges with adherence** Remembering a time that I had my coach session. Just because of the fatigue that I have. It throws my day all over the place sometimes. Because sometimes I get tired, sometimes I forget (the session) because I’m so tired. (ID #83)
Long SSc duration (*n* = 10)	Improved	**Re-established healthy routines** I felt like kind of getting back into the better routines and eating a little bit better and doing those things really did, you know, over the course of a couple of weeks, improve the fatigue level dramatically. (ID #34)
	Limited improvement	**Skipped modules** I really didn’t get into a lot of the modules…I’m not a meditating kind of guy. (ID #144)
		**Seeking fresh content** Some of us aren’t new to scleroderma, so that can get boring for us because we’ve seen it 50 times. So it’s like, you know, just thinking of having something fresh content for people that have had it. (ID #24)
		**SSc-specific symptoms content is needed** You should cover like a chapter on all of those with the Sjogren’s, the Raynaud’s, with the esophagus problems, with the scar tissue internally, with your lungs and your bowels. (ID #115) Some of the stuff like some of the video things that I watched, it wasn’t, there wasn’t a lot of new information. So I think I could have learned more with you know, a little bit more in depth of…joint mobility or something. (ID #65)
		**Challenges with adherence** Remembering to do my goals, not forgetting about them. (ID #24) I have high stress because I’m responsible for everything in my household. That is a problem, I think I touched on this with my coach, but you know I’m responsible if the meals don’t go right I get heck for my husband. I get heck on a lot of things…and it’s really frustrating, it is really stressful. (ID #29)
Theme: Easy program access through technology		
Short SSc duration (*n* = 11)	Improved	**Convenience of technology** It’s not better than face to face in person, but at least it’s still face to face with somebody, you know. And being able to have that was a huge bonus to keep my head in the right space. (ID# 93) Having a chance to go back and revisit some of these topics now that you’ve been through the whole entire program. (ID #73) **App is not easy to track goals** I think tracking (on the app) was kind of hard. It was little, you know, clunky, and making selections, and it just kind of the flow of it. (ID #135)
	Limited improvement	**App is challenging to use** I think if the app had been a little different maybe that would have been easier for me. But yeah I wouldn’t even necessarily say that’s a negative beyond the app, it’s just that was a challenge for me. (ID #107)
Long SSc duration (n = 10)	Improved	**App is not easy to navigate** It wasn’t user friendly. It wasn’t easy to navigate. (ID #142)
	Limited improvement	**Website is user-friendly** The website and different modules, they are easy to navigate. And I thought that was really helpful, because it wasn’t difficult for me to find a topic and get the content (ID #24). **App needs improvement** The app needs some help because I get lost in there. (ID #144)

Regarding the need for a tailored approach, most participants in the short/improved group were satisfied with the current program, while a few expressed the need for slower pacing of the program, as weekly coaching sessions could be overwhelming for individuals who were still processing their diagnosis and had to attend many doctor’s appointments. Another participant suggested pairing participants with a health coach with similar symptom severity to enable peer health coaches to provide health goals that align more easily with the participants’ experiences. Conversely, two participants in the short/limited improvement group faced challenges in program adherence due to severe fatigue, resulting in forgetting to attend sessions. In the long/improved group, a participant reported participating in RENEW helped re-establish a healthy routine. However, others in the long/limited improvement group found certain modules irrelevant and chose to skip them. These participants, who had SSc for >5 years, emphasized the need for more content addressing specific SSc symptoms, such as dry mouth/eyes and joint mobility. They also reported challenges to program adherence due to daily stressors, leading them to forget to complete weekly healthy goals. Regarding the use of technology, participants in all groups appreciated the convenience but had suggestions for improvement, particularly regarding the functionality of setting and tracking health goals in the app.

## Discussion

This is the first mixed methods study to examine participants’ experiences of a resilience-building energy management intervention on symptoms and well-being in people with SSc. Qualitative analysis was conducted to further explore the experiences of participants in the RENEW intervention program. The quantitative and qualitative findings were integrated in this study to provide nuanced insights into how the duration of SSc influences participants’ experiences of fatigue.

Qualitative results underscored the importance of establishing a meaningful relationship between participants and their peer health coaches in RENEW. This connection was a key feature contributing to the program’s effectiveness. The inclusion of peer health coaches in RENEW provided participants with accountability and personalized guidance that can be challenging to achieve when progressing through the program alone [15]. These peer health coaches, who also have SSc, not only provided support, but also served as role models who demonstrated positive health behaviours, which in turn motivated participants to continue working towards their goals. Our study builds upon previous research, demonstrating the positive impact of such supportive figures on participants’ health outcomes [16–18]. Specifically, our qualitative study highlighted the significance of social support derived from the bonding and interactions between participants and their peer health coaches. Prior research has shown that individuals receiving support from peers exhibit better adherence to medication [45] and report enhanced physical and psychological well-being [16–19]. Our findings reinforce the impact of the participant–coach relationship in SSc symptom management programs and the significant role that peer health coaches can play in fostering an impactful intervention program for achieving health goals.

While quantitative results showed a clinically significant reduction in fatigue [32], our qualitative data revealed specific preferences and suggestions for enhancing the potential effectiveness of RENEW. Varied preferences for coaching session frequency underscore the need for flexibility and individualization in healthcare for individuals with SSc, particularly with supported self-management interventions. Additionally, the desire for continued support post-program highlights potential value for those facing geographical or SSc resource challenges. Some participants reported no or limited improvement in SSc-specific symptoms, underscoring the complexity of SSc, as it can manifest with a wide range of symptoms that vary individually. There may be aspects of symptom experiences that require specific attention and support, indicating a need for intervention programs that can adapt to participants’ evolving SSc-related symptoms and challenges over the years. Given the complexity and severity of SSc, implementing education programs alongside peer health coaching could be a beneficial strategy for improving access to, understandability and adoption of this critical information. Notably, our international participants highlight the need for considering cultural and geographical differences in healthcare interventions. It is essential to recognize that the availability of resources, treatment options and even the terminologies used to describe SSc can vary from one region, state and country to another. This divergence can lead to challenges for individuals seeking SSc-related information, especially when resources are primarily tailored to a specific region. Addressing this issue requires a more patient-centred, inclusive approach to the development of interventions and resources for individuals with SSc. Collaborating with individuals with SSc, experts and organizations from across the world can help to ensure relevance and accessibility to individuals regardless of location.

The use of technology was an advantage according to participants. The convenience offered by our website and app was evident and in line with the broader trend of integrating technology into SSc interventions [15, 46, 47]. The use of digital tools for information dissemination and video calls for coaching sessions was particularly valued by participants. These tools provided easy access from the comfort of participants’ homes, reducing logistical challenges like transportation and scheduling conflicts [48]. However, participants also provided feedback concerning the app’s usability, including aspects such as navigation and the goal-setting layout. Addressing these suggestions in future app development has the potential to enhance participants’ overall experiences.

The mixed methods analysis revealed similarities and differences among RENEW participants relative to improvement in fatigue. Those experiencing significant reductions in fatigue shared common experiences, including increased self-awareness of SSc-related symptoms, better day planning and recognizing the need for breaks. Additionally, participants mentioned acquiring stress management and relaxation techniques, aligning with previous research findings from symptom management interventions in people with rheumatic diseases [23, 24, 49]. Nonetheless, differences emerged concerning tailoring of the program based on SSc duration. Participants with short SSc duration preferred a slower-paced program, reflecting their need to digest SSc-related information. Bi-weekly coaching sessions focusing on fundamental aspects of symptom management such as activity pacing and lifestyle adjustments may be beneficial. Additionally, pairing these participants with peer health coaches experiencing similar symptom severity could facilitate more personalized and productive conversations between participants and health coaches. In contrast, participants with long SSc duration sought more SSc-specific symptom management information, focusing on issues like dry mouth/eyes, gastrointestinal problems or joint mobility. Tailoring the program with SSc-related content could enhance participants’ ability to effectively manage symptoms associated with long-standing SSc.

Future research could focus on individualizing the RENEW intervention based on SSc duration and symptom severity to maximize program effectiveness. Additionally, further studies are needed to examine the long-term impacts of RENEW beyond the 12-week study period. Such investigations could provide insights into RENEW’s sustainability and uncover delayed or cumulative effects that may not be apparent in short-term assessments. We acknowledge the need for app modification and plan to improve it based on participants’ feedback. Leveraging the convenience of technology, future studies could examine the differences between asynchronous and synchronous communication within the intervention. By evaluating which communication approach is more effective (e.g. pre-recorded videos made by peer health coaches *vs* real-time video interactions with a health coach), researchers can gain a deeper understanding of the most effective approach for symptom management in people with SSc.

This study had some limitations. While male and racial/ethnic minority participants were underrepresented in our intervention, we tried to address this issue through purposeful sampling to include a higher proportion of underrepresented participants in the interview than the proportion completing the intervention. This approach was used to achieve more generalizable results. More than 60% of our participants have diffuse SSc, which may limit the applicability of the study results to the broader SSc population. The absence of SSc clinical manifestations such as digital ulcers, interstitial lung disease and joint involvement limits our ability to understand how disease duration could impact SSc-specific symptoms and fatigue. Using technology to deliver interventions may limit generalizability, as it excluded participants who may be unable to purchase, access or use the technology required. Major strengths of this study are noteworthy. First, the use of a mixed methods analysis is particularly significant, especially within the rheumatology field, where such studies are relatively scarce. The integration of quantitative and qualitative findings provided additional insights into the impact of SSc duration on fatigue. Such information is enlightening and can help us to make meaningful refinements to RENEW. Second, we actively involved patient partners from the development to the implementation of the RENEW program. This ensured that the intervention was designed to meet the specific needs of the SSc population. Third, the virtual coaching sessions addressed physical limitations and logistical constraints (e.g. time to get to in-person appointments) often faced by individuals with SSc.

Our study highlights the importance of the participant–peer health coach relationship and tailoring interventions to the unique needs of participants based on their SSc duration and symptom severity. A one-size-fits-all approach will not fully address the diverse preferences and needs of the SSc population. The use of technology in RENEW offered convenience and flexibility, yet also presented opportunities for refinement. Future studies should work on program refinement and evaluating the long-term effects and different communication approaches to further examine RENEW’s impact on fatigue and symptom management.

## Data Availability

The data underlying this article will be shared upon reasonable request to the corresponding author.
